# Evaluation of Galanin Expression in Colorectal Cancer: An Immunohistochemical and Transcriptomic Study

**DOI:** 10.3389/fonc.2022.877147

**Published:** 2022-05-30

**Authors:** Iman M. Talaat, Nada M. Yakout, Ahmed S.A. Soliman, Thenmozhi Venkatachalam, Arya Vinod, Leen Eldohaji, Vidhya Nair, Amal Hareedy, Alaa Kandil, Wael M. Abdel-Rahman, Rifat Hamoudi, Maha Saber-Ayad

**Affiliations:** ^1^ Department of Clinical Sciences, College of Medicine, University of Sharjah, Sharjah, United Arab Emirates; ^2^ Sharjah Institute for Medical Research, University of Sharjah, Sharjah, United Arab Emirates; ^3^ Pathology Department, Faculty of Medicine, Alexandria University, Alexandria, Egypt; ^4^ Pathology Department, National Research Center, Cairo, Egypt; ^5^ Department of Physiology and Immunology, College of Medicine and Health Science, Khalifa University, Abu Dhabi, United Arab Emirates; ^6^ Pathology Department, Faculty of Medicine, Cairo University, Cairo, Egypt; ^7^ Clinical Oncology and Nuclear Medicine Department, Faculty of Medicine, Alexandria University, Cairo, Egypt; ^8^ Department of Medical Laboratory Sciences, College of Health Sciences, University of Sharjah, Sharjah, United Arab Emirates; ^9^ Division of Surgery and Interventional Science, University College London, London, United Kingdom; ^10^ Pharmacology Department, Faculty of Medicine, Cairo University, Cairo, Egypt

**Keywords:** galanin, immunohistochemistry, transcriptomic analysis, GALR1, colorectal cancer, bioinformatics, TIMER 2

## Abstract

Colorectal cancer (CRC) represents around 10% of all cancers, with an increasing incidence in the younger age group. The gut is considered a unique organ with its distinctive neuronal supply. The neuropeptide, human galanin, is widely distributed in the colon and expressed in many cancers, including the CRC. The current study aimed to explore the role of galanin at different stages of CRC. Eighty-one CRC cases (TNM stages I – IV) were recruited, and formalin-fixed paraffin-embedded samples were analyzed for the expression of galanin and galanin receptor 1 (GALR1) by immunohistochemistry (IHC). Galanin intensity was significantly lower in stage IV (n= 6) in comparison to other stages (p= 0.037 using the Mann-Whitney U test). Whole transcriptomics analysis using NGS was performed for selected samples based on the galanin expression by IHC [early (n=5) with high galanin expression and late (n=6) with low galanin expression]. Five differentially regulated pathways (using Absolute GSEA) were identified as drivers for tumor progression and associated with higher galanin expression, namely, cell cycle, cell division, autophagy, transcriptional regulation of TP53, and immune system process. The top shared genes among the upregulated pathways are *AURKA, BIRC5, CCNA1, CCNA2, CDC25C, CDK2, CDK6, EREG, LIG3, PIN1, TGFB1, TPX2*. The results were validated using real-time PCR carried out on four cell lines [two primaries (HCT116 and HT29) and two metastatic (LoVo and SK-Co-1)]. The current study shows galanin as a potential negative biomarker. Galanin downregulation is correlated with advanced CRC staging and linked to cell cycle and division, autophagy, transcriptional regulation of TP53 and immune system response.

**Graphical Abstract:**
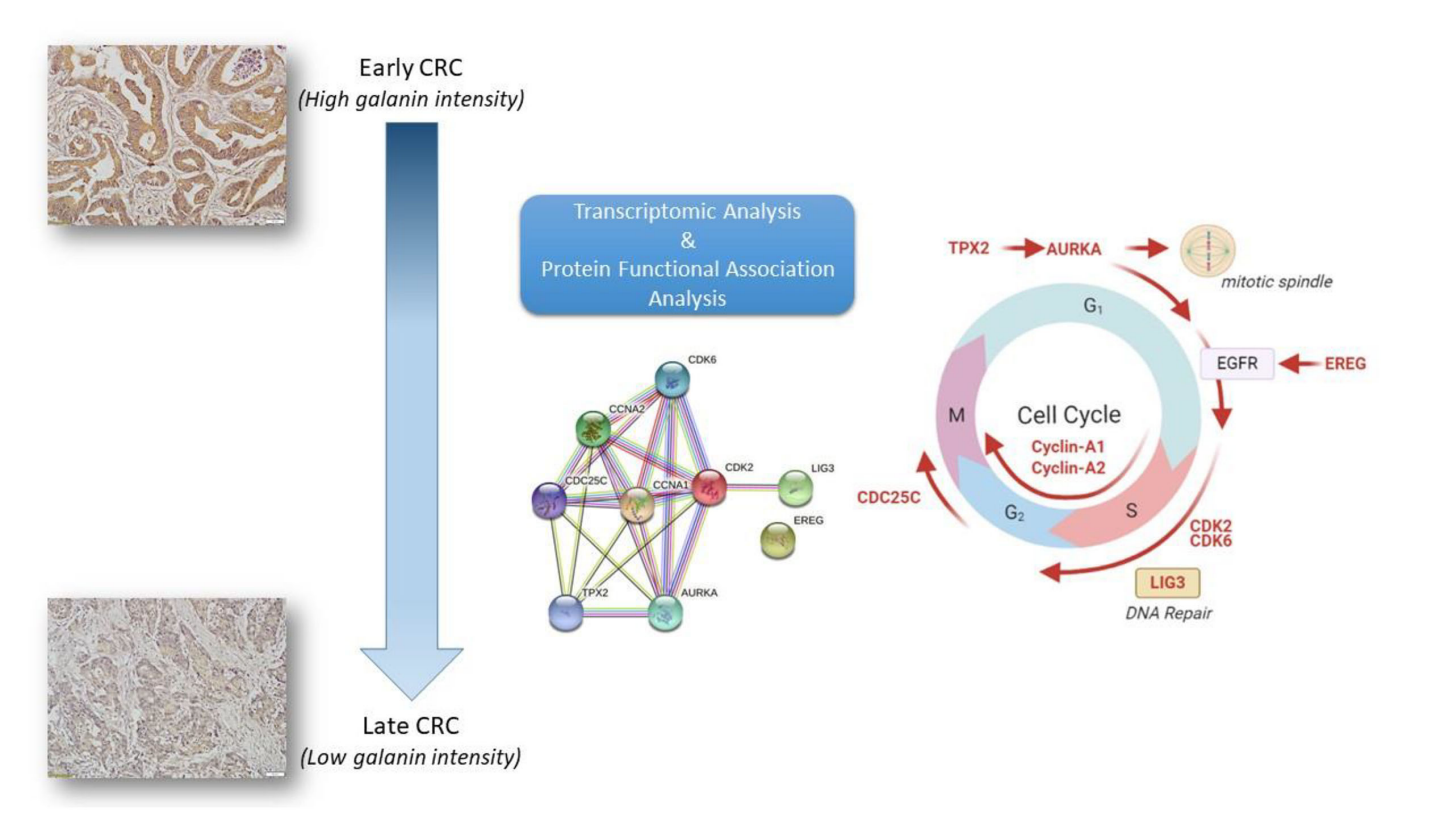


## Introduction

Colorectal cancer (CRC) is the third most common cancer in men and the second in women worldwide, representing 9.7% of all cancers (excluding non-melanoma skin cancer) (1). It is the second leading cause of cancer-related mortality in Europe and North America, with a global 900,000 deaths every year. The incidence has markedly accelerated in the Middle East and North Africa (8.8 in 1990 to 12.4 years in 2017) with 39.9%, most likely due to adopting the western lifestyle (2). CRC biomarkers have been extensively studied in Caucasians, but only a few studies have been conducted on the North African population.

Intriguingly, the gut is considered a unique organ with its distinguished hormonal and neuronal supply, highlighting the significance of studying the role of its neuropeptides. Human galanin is a 30-amino acid neuropeptide widely distributed in the central nervous system, heart and skin (3). The expression of galanin has been observed in many tumors. The level of expression was shown to correlate with differentiation or aggressiveness of the tumor (4). Galanin peptide is highly expressed in human pheochromocytoma, the first tumor in which galanin was identified (3, 5). It is also expressed in the pituitary adenoma (3, 6), gangliocytoma (7), neuroblastoma (8), and paraganglioma (9). Galanin was also detected in melanoma, small cell lung carcinoma, several breast cancers and head and neck squamous cell carcinoma and basal cell carcinoma (3, 4, 10). In 2007, Kim et al. showed that galanin is upregulated in colon cancer in Korean patients (11). Another study showed that high expression of galanin was associated with poor prognosis in stage II CRC in Japanese patients, as it was associated with higher invasiveness of cancerous cells. Galanin works in an autocrine and/or paracrine approach on G-protein coupled receptors. Galanin receptors are believed to be implicated in tumor progression, epithelial-mesenchymal transition (EMT) and chemotherapeutic sensitivity of colorectal cancer (12).

The aim of the current study was to quantify the expression of galanin and GALR1 in human tissue samples of Egyptian patients at different stages of CRC, and to correlate the results with various clinicopathological parameters. In addition, a subset of well-characterized samples from the early and late stages of CRC was subjected to transcriptomics analysis to explore the differentially expressed genes and pathways. Furthermore, the transcriptomics results were validated using primary and metastatic CRC cell lines.

## Patients And Methods

### Patients and Sample Recruitment

The formalin-fixed paraffin-embedded (FFPE) tumor blocks of primary CRC were surgically managed in Alexandria & Cairo University Main Hospitals. Clinicopathological data of the patients along with the available follow-up data were retrieved from the medical records. The studied clinicopathological variables included age, gender, location, size, shape, histopathological variant, grade, depth of invasion (T), number of lymph node metastases (N), distant metastases (M), TNM staging, and state of the surgical margins. The present work was approved by the Research Ethics Committee (REC), Alexandria University and the University of Sharjah (Ref. No.: REC-17-11-23-01). The study was conducted according to the principles of the Declaration of Helsinki of 1975 (revised 1983). As this study was retrospectively conducted on FFPE blocks, the need for patients’ consent was waived by the REC.

### Histopathological Examination

The FFPE blocks of 81 cases diagnosed as primary CRC were cut into 4-μm thick sections and stained by Hematoxylin and Eosin, followed by microscopic examination. Two independent pathologists evaluated the histopathological parameters. The histologic type was assessed according to the 2019 WHO classification, and the staging was performed according to the TNM staging (8^th^ edition).

### Immunohistochemical Methods

In total, 81 samples were analyzed for the expression of galanin and galanin receptor 1 (GALR1) by immunohistochemistry (IHC). Manual immunostaining was performed on 4-μm-thick sections as described previously (13). In brief, FFPE sections were deparaffinized in xylene, rehydrated in graded alcohol, immersed in 0.01 M citrate buffer (pH 6.0), and heated in a domestic microwave oven at full power for 2x5 minutes and left in buffer to cool at room temperature. The sections were then incubated in 0.3% hydrogen peroxide for 20 minutes to block endogenous peroxidase activity. Incubation with the primary antibodies [Anti-GAL (ab216399), and anti-GALR1 (ab150486); Abcam, Cambridge, UK] at a concentration of 1:200 diluted in 1% bovine serum albumin/tris-buffered saline was carried out overnight in a humid chamber at 4°C according to the manufacturer’s instructions. The following day, the slides were washed and incubated first with biotinylated secondary antibody (SignalStain^®^ Boost IHC Detection Reagent; Cell Signaling Technology) for 30 min at 20°C, then with avidin-biotin-peroxidase complex for 30 min at 20°C (Vectastain ABC kit; Abcam, Cambridge, UK). For visualization, the peroxidase/DAB DAKO Real ENVision detection system (DAKO, Glostrup, Denmark) was used, following the manufacturer’s instructions. For each run of IHC staining, positive and negative control sections were included. Human skin sections (epidermis, sweat glands) were used as positive controls for GAL (14, 15). Moreover, galanin expression in Meissner’s submucosal plexus in the colon and GALR-1 expression in immune cells in the colonic mucosa were considered internal positive controls (13).

### Immunostaining Assessment

The immunoreactivity of galanin was assessed in the cancer cells based on the staining intensity (SI) and the percentage of positively stained cells (PP) to create the immunoreactive score (IRS) as follows: IRS=SI x PP, for each sample, as previously described (16). The intensity was scored as follows: 0: no staining, 1: weakly positive, 2: moderately positive and 3: strongly positive. The percentage of stained tumor cells was estimated by excluding the adjacent normal-appearing tissue, as well as necrotic or hemorrhagic foci if present and it was scored as follows: 0: 0%, 1: 0-9%, 2: 10-49% and 3: 50-100%. Accordingly, the IRS score ranged from 0 to 9, designated as a low expression for a score of 0 to 3, and a high expression for a score of 4 to 9 (5). A Semi-quantitative evaluation of the immunostained slides was performed blindly and independently by the pathologists, using an Olympus microscope (BX51; Olympus, Tokyo, Japan). All discordant cases were resolved within consensus meetings.

### Whole Transcriptomics Analysis

Tissue curls were cut from FFPE blocks for representative samples from each category high and low galanin intensity as per IHC interpretation, and according to TNM staging [stage I (n=5), stage II (n=4) and stage III (n=2)]. The samples were classified into early (stage I) and late (stage II and III).

The tissue sections were subjected to RNA extraction using RecoverAll™ Total Nucleic Acid Isolation Kit for FFPE (Invitrogen) using the manufacturer’s instruction. The extracted RNA was further purified and concentrated using Zymo RNA clean and concentrator kit (Zymo Research, USA). Thus, purified RNA was quantified using Qubit 3 fluorometer (Invitrogen) and ~50ng of RNA was sequenced using Ion Ampliseq transcriptome panel on S5 XL System (ThermoFisher) capturing more than 21,000 transcripts. In brief, ~30ng of Turbo DNase treated RNA was used for cDNA synthesis using SuperScript VILO cDNA Synthesis kit (Invitrogen) followed by amplification using Ion AmpliSeq gene expression core panel primers. The prepared library was purified using Agencourt AMPure XP Beads (Beckman Coulter) and the purified library was quantified using Ion Library TaqMan™ Quantitation Kit (Applied Biosystems). The libraries were further diluted to 100pM and pooled equally with four individual samples per pool. The pooled libraries were amplified using emulsion PCR on Ion OneTouch™ 2 instrument (OT2) and the enrichment was performed on Ion OneTouch™ ES following manufacturer’s instruction. Thus, prepared template libraries were then sequenced with an Ion S5 XL Semiconductor sequencer using the Ion 540™ Chip.

Five early (stage I) cases of CRC showing low galanin intensity on IHC and six late cases (4 ‘stage II’ and 2 ‘stage III’) showing a high intensity of galanin were subjected to transcriptomics analysis.

### Bioinformatics Analysis

RNA-seq data were analyzed using Ion Torrent Software Suite version 5.4 and the alignment was carried out using a modification of the Torrent Mapping Alignment Program (TMAP), optimizing it for aligning the raw sequencing reads against reference sequence derived from hg19 (GRCh37) assembly. The specificity and sensitivity were maintained by implementing a two-stage mapping approach by employing BWA-short, BWA-long, SSAHA (17), Super-maximal Exact Matching (18) and Smith-Waterman algorithm (19) for optimal mapping. Raw read counts of the targeted genes were performed using Sam tools (Sam tools view –c –F 4 –L bed_file bam_file) and the number of expressed transcripts was confirmed after Fragments Per Kilobase Million (FPKM) normalization. Differentially expressed gene (DEG) analysis was performed using an in-house script written in R programming language (version 3.6.3) with function calls to the DESeq2 package from Bioconductor libraries. Raw read counts from RNA Seq were subjected to quantile normalization and transcript counts ranked below 1 were excluded. Differentially expressed genes between the two sets of tissue samples [early-stage CRC with a high galanin intensity (n=5), and late stages with a low galanin intensity (n=6)] were assessed using a 2-tailed t-test. Differentially expressed genes with a p-value of <0.05 and a 5-fold difference were included for pathway analysis using Metascape.

Absolute GSEA was carried out as previously described (20). An R script to calculate the frequency of the gene recurrence across pathways was used to curate the shared genes among the top 5 differentially expressed pathways of interest. We further investigated the status of the resultant genes in COAD TCGA PanCancer datasets (n= 592). We used Gene Set Cancer Analysis (GSCALite), a web tool that provides a prediction of the gene activity in the cancer-associated pathway, among other functions through the website: http://bioinfo.life.hust.edu.cn/web/GSCALite/, and http://bioinfo.life.hust.edu.cn/GSCA/#/expression (accessed on 14 April 2022) (21),. We studied the galanin expression in publicly available cancer data sets from CPTAC (n=197) and TCGA (n=194), to compare normal versus CRC specimens as well as the expression in different age groups, respectively. We used UALCAN, a web-based tool that uses OMICS data (http://ualcan.path.uab.edu accessed on 14 April 2022) (22).

### Cell Lines

To validate the transcriptomics results, we used 4 human colorectal adenocarcinoma cell lines, namely; HCT116, HT29, SK-Co-1, and LoVo. HCT116 and HT29 are primary CRC cell lines, LoVo cells are derived from a metastatic lymph node, whereas Sk-Co-1 cells are derived from ascitic fluid from a patient with CRC. HCT116 and HT29 were maintained in RPMI. SK-CO-1 and LoVo were maintained in Minimum Essential Media, and Dulbecco’s Modified Eagle’s Medium Nutrient Mixture F-12 Ham respectively. Both types of media were supplemented with 10% fetal bovine serum and 1% antibiotics (penicillin/streptomycin). Cells were cultured in 75-cm^2^ tissue culture flasks and incubated in a humidified incubator at 37°C in 5% CO_2_ and 95% room air and sub-cultured every 3-4 days with trypsin.

### Real-Time PCR

Total RNA from the cell lines was extracted using PureLink^®^ RNA Mini Kit (ThermoFisher, USA) according to the manufacturer’s instructions and quantified using Nanodrop (Invitrogen) A total of 500 ng RNA was reverse transcribed using High-Capacity cDNA Reverse Transcription Kit and Real-time PCR was then performed using 5X Firepol sybergreen master mix (Solis Biodyne, Estonia) on Quantstudio3 (Thermo, USA). Relative quantification was expressed as 2^−ΔΔ^ *^C^* ^t^, where ΔΔ*C*t is the difference between the Δ*C*t values of the control and the starved cells and the samples analyzed in triplicates. The 18s rRNA gene was used as endogenous control and all the primers used in the RT–qPCR assays are listed in **Table 1**.

**Table 1:** List of primers used in real-time PCR.

Gene	Primer (5’-3’)	Primer (3’-5’)	Amplicon size (bp)
**AURKA**	GCAACCAGTGTACCTCATCCTG	AAGTCTTCCAAAGCCCACTGCC	158
**BIRC5**	CCACTGAGAACGAGCCAGACTT	GTATTACAGGCGTAAGCCACCG	115
**CCNA1**	GCACACTCAAGTCAGACCTGCA	ATCACATCTGTGCCAAGACTGGA	118
**CCNA2**	CTCTACACAGTCACGGGACAAAG	CTGTGGTGCTTTGAGGTAGGTC	120
**CDC25C**	AGAAGCCCATCGTCCCTTTGGA	GCAGGATACTGGTTCAGAGACC	133
**CDK2**	ATGGATGCCTCTGCTCTCACTG	CCCGATGAGAATGGCAGAAAGC	97
**CDK6**	GGATAAAGTTCCAGAGCCTGGAG	GCGATGCACTACTCGGTGTGAA	106
**EREG**	CTCTACACAGTCACGGGACAAAG	CTGTGGTGCTTTGAGGTAGGTC	120
**LIG3**	GCTACTTCAGCCGCAGTCTCAA	GCAGTGGTTTGCCTGTCTTGTTG	147
**PIN1**	ACAGTTCAGCGACTGCAGCTCA	GCAGCGCAAACGAGGCGTCTT	101
**TGFB1**	TACCTGAACCCGTGTTGCTCTC	GTTGCTGAGGTATCGCCAGGAA	122
**TPX2**	TTCAAGGCTCGTCCAAACACCG	GCTCTCTTCTCAGTAGCCAGCT	131

Real-time PCR was carried out on Quant Studio 3 (Thermo Fisher) using HOT FIREPol^®^ EvaGreen^®^ qPCR Supermix. 18S was used as the housekeeping gene. The PCR reaction mixture and cycling conditions were performed according to manual instructions. All samples were amplified in triplicates. The average threshold cycle (Ct) values were obtained from each reaction and the gene expression was quantified using the 2(−ΔΔC(T)) relative method.

### Statistical Analysis

Testing for normality was performed for continuous variables. When data were not normally distributed, the non-parametric Mann-Whitney U test was used to evaluate the significant differences between groups of samples, and to compare gene expression of different cell lines. Pearson Correlation was used to identify possible correlations of galanin intensity with various clinicopathological features; p <0.05 denoted a significant difference. Adjusted p < 0.8 was used as a cutoff point for identifying the differentially expressed genes in the transcriptomics analysis. SPSS version 26 was used to perform statistical analysis (SPSS Inc, IBM, Chicago, Illinois).

## Results

### Clinicopathological Data

In total, 81 CRC cases were recruited, with a mean age of 53.19 ± 13 years (range 22-86); 52 female patients and 29 male patients with a female: male ratio of 1.7:1. All specimens were diagnosed as adenocarcinoma (NOS) except for one case diagnosed as mucinous adenocarcinoma (not graded based on the degree of glandular differentiation) (23, 24). The clinicopathological data is shown in **Supplementary Table S1**. Staging of cancer showed significant negative correlation with the patients’ age (p = 0.006, r = -0.305). Other parameters did not show significant correlations (**Supplementary Table S2**).

### Galanin Intensity and TNM Staging

**Figure 1:**
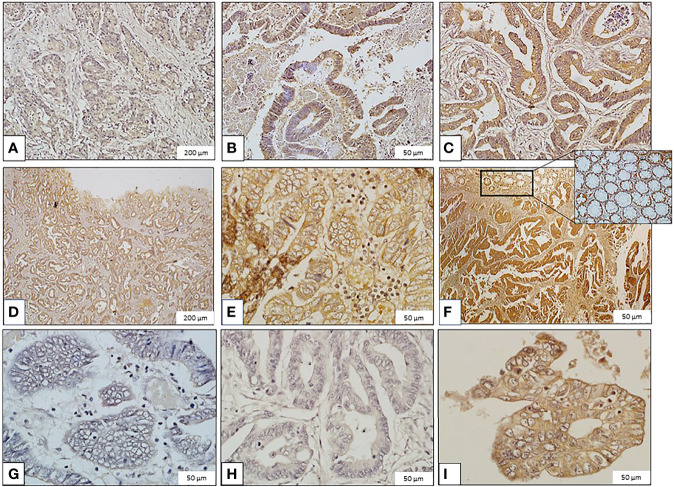
Images of immunohistochemical staining of galanin in colorectal carcinoma (CRC) samples (Immunoperoxidase, scale bar: 200μm ‘panels **A**, **D**’ and 50μm ‘panels **B**, **C**, **E**, **F**–**I**’). Galanin Expression: **(A)** Mild expression in grade II, stage I CRC. **(B)** Moderate expression in grade II, stage I CRC. **(C)** High expression in grade II, stage II CRC. **(D)** Mild expression in grade I, stage III CRC. **(E)** Moderate expression in grade I, stage III CRC. **(F)** High expression in grade I, stage II CRC. The inset shows galanin expression in normal colonic mucosa. **(G)** Minimal expression in grade III, stage IV CRC. **(H)** Mild expression in grade II, stage IV CRC. **(I)** Moderate expression in grade II, stage IV CRC.

Immunohistochemical analysis of the 81 CRC samples revealed focal and diffuse galanin immunoreactivity in the majority of cases (**Figure 1**). Twenty-one cases revealed no expression, 28 showed mild, 22 were moderate, whereas only 10 cases revealed intense galanin expression. GALR1 expression generally showed the same trend in the CRC samples (**Supplementary Figure S1A** shows images of immunohistochemical staining of Galanin Receptor 1 (GALR1). **Supplementary Figure S1B** is a bar graph representing GALR1 intensity according to TNM staging). **Supplementary Table S3** shows the galanin intensity across the different TNM stages.

**Figure 2:**
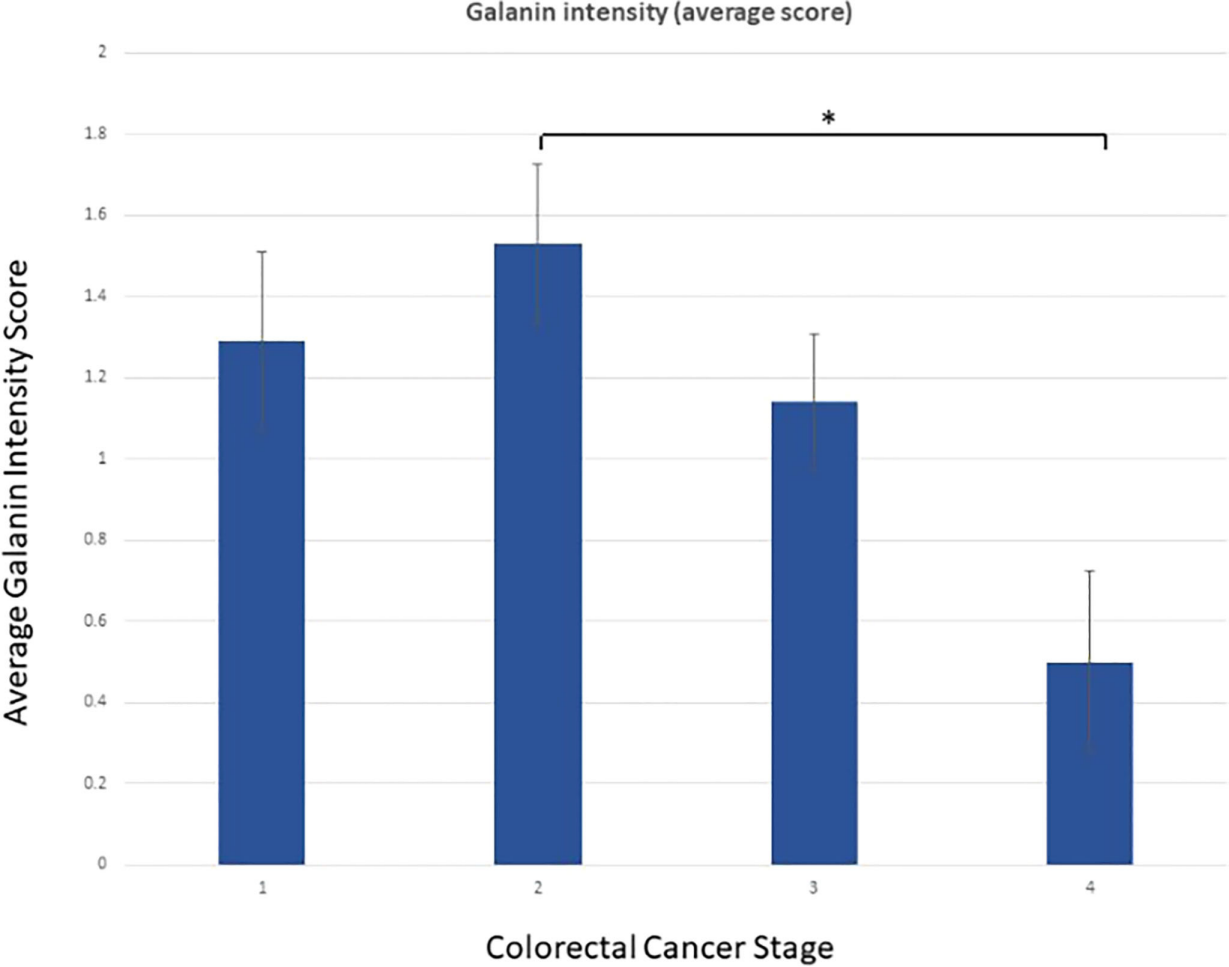
Galanin intensity according to TNM staging. Galanin intensity is significantly lower in stage IV CRC compared to stage I-III (p= 0.037, using the non-parametric Mann-Whitney U test). * p-value <0.05.

Galanin intensity showed a trend of negative correlation with TNM staging of the tumors (P-value =0.054, Pearson correlation of -0.215). The correlation with individual parameters revealed a negative correlation of galanin intensity with the “number of infiltrated lymph nodes” score (p-value <0.001, Pearson correlation of -0.384). N0: no lymph node metastasis, N1: 1-3 metastasis to regional lymph nodes, N2: metastasis to 4 or more regional lymph nodes, and with “distant organ metastasis” score (p-value of 0.039, Pearson correlation of -0.230), (0, 1 for absent or present distant metastasis, respectively). Galanin intensity was significantly lower in stage IV (n= 6) in comparison to other stages (p= 0.037, using the Mann-Whitney U test), **Figure 2**.

### Transcriptomics Analysis

**Figure 3:**
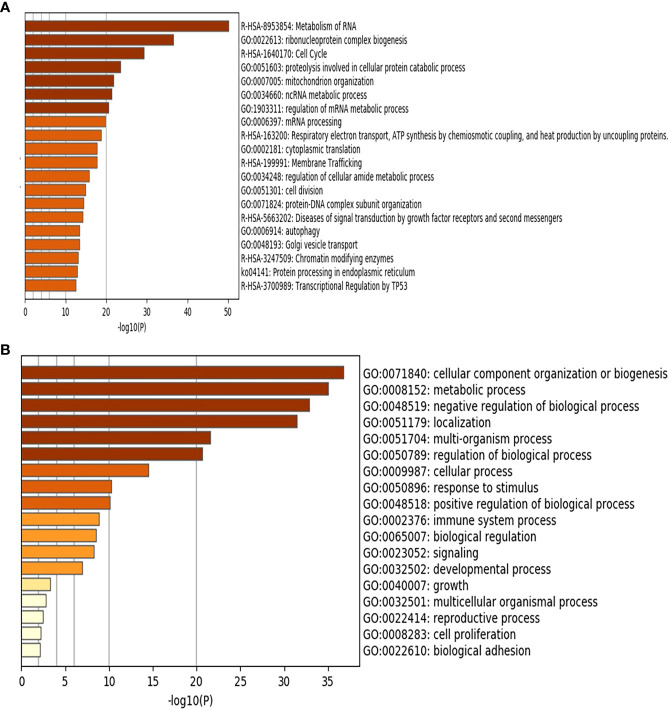
Gene Set Enrichment Analysis (GSEA) of the differentially expressed genes showing the upregulated pathways in late stages of CRC (n=6) compared to early stages (n=5) using Metascape (http://metascape.org): a gene annotation and analysis online resource generating a graphical presentation. **(A)** Using Reactome and KEGG databases. **(B)** Using GO database.

Whole transcriptomics analysis using NGS captured more than 21,000 transcripts, with 1337 differentially expressed genes. Gene Set Enrichment Analysis (GSEA) was performed as previously described (20). An R-script was used to calculate the frequency of the gene recurrence across cellular pathways. Differentially upregulated pathways in late CRC stages (TNM stage II and III with high galanin intensity) compared to early-stage (TNM stage I with low galanin intensity) are shown in **Figure 3**. The upregulated genes are members of five key pathways involved in CRC, namely cell cycle (170 genes), cell division (603 genes), autophagosome (40 genes), and transcriptional regulation by TP53 (362 genes) and immune system process (332 genes). The supplementary figures (**Supplementary Figures S2**–**S8**) show the normalized differential expression of different pathways.

**Figure 4:**
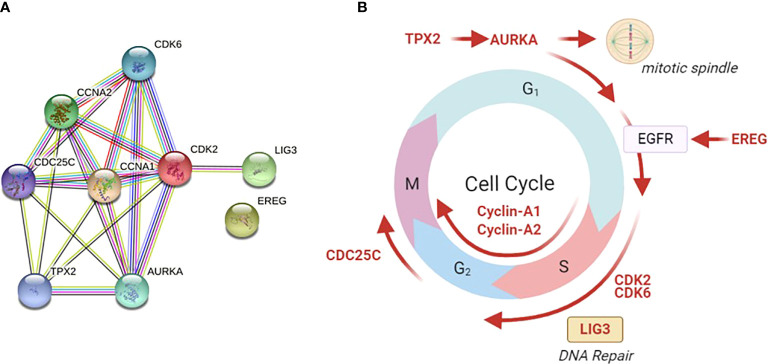
Functional protein analysis of the most frequently shared genes among upregulated pathways in late versus early CRC stages. The analysis was performed using the STRING functional protein association network. **(A)** Functional protein analysis was performed for the most frequently shared kinases appearing twice in upregulated pathways in late versus early CRC stages (using STRING functional protein association network). TPX2 mediates AURKA localization to spindle microtubules. CDKs = cyclin-dependent kinases. AURKA = Aurora kinase A, EREG = Proepiregulin; Ligand of the EGF receptor/EGFR and ERBB4, LIG3 = DNA ligase 3, TGFβ = Transforming growth factor beta-1, TPX2 = Targeting protein for Xklp2. **(B)** The site of action of each gene is shown in different phases of the cell cycle. Galanin was not linked to any of those genes in the STRING functional protein association network.

The top shared genes among the upregulated pathways are *AURKA, BIRC5, CCNA1, CCNA2, CDC25C, CDK2, CDK6, EREG, LIG3, PIN1, TGFB1, TPX2* (**Table 2**). Functional protein analysis was performed to get more insight into the relationship among different coded proteins (**Figure 4**).

**Table 2:** Most frequently shared genes among top differentially upregulated pathways in late CRC.

Gene	Annotation
*AURKA*	Aurora kinase A; Mitotic serine/threonine kinase that contributes to the regulation of cell cycle progression. It plays a critical role in various mitotic events including the establishment of mitotic spindle, centrosome duplication, centrosome separation as well as maturation, chromosomal alignment, spindle assembly checkpoint, and cytokinesis.
*BIRC5*	Baculoviral IAP repeat containing 5 (microtubule cytoskeleton organization). BRIC5 is also a member of the inhibitor of apoptosis gene family.
*CCNA1*	Cyclin-A1; is involved in the control of the cell cycle at the G1/S (start) and G2/M (mitosis) transitions. it belongs to the cyclin family.
*CCNA2*	Cyclin-A2; Cyclin which controls both the G1/S and the G2/M transition phases of the cell cycle. Functions through the formation of specific serine/threonine protein kinase holoenzyme complexes with the cyclin-dependent protein kinases CDK1 or CDK2.
*CDC25C*	M-phase inducer phosphatase 3; Functions as a dosage-dependent inducer in mitotic control. Tyrosine protein phosphatase required for progression of the cell cycle.
*CDK2*	Cyclin-dependent kinase 2; Serine/threonine-protein kinase involved in the control of the cell cycle; essential for meiosis, but dispensable for mitosis. It triggers duplication of centrosomes and DNA. Acts at the G1-S transition to promote the E2F transcriptional program and the initiation of DNA synthesis, and modulates G2 progression.
*CDK6*	Cyclin-dependent kinase 6; Serine/threonine-protein kinase involved in the control of the cell cycle and differentiation; promotes G1/S transition. Phosphorylates pRB/RB1 and NPM1.
*EREG*	Proepiregulin; Ligand of the EGF receptor/EGFR and ERBB4. Stimulates EGFR and ERBB4 tyrosine phosphorylation. Contributes to inflammation, wound healing, tissue repair, regulating angiogenesis.
*LIG3*	DNA ligase 3; Isoform 3 functions as heterodimer with DNA-repair protein XRCC1 in the nucleus and can correct defective DNA strand- break repair and sister chromatid exchange following treatment with ionizing radiation and alkylating agents.
*PIN1*	Peptidyl-prolyl cis-trans isomerase NIMA-interacting 1; Peptidyl-prolyl cis/trans isomerase (PPIase) that binds to and isomerizes specific phosphorylated Ser/Thr-Pro (pSer/Thr- Pro) motifs. - Regulates mitosis presumably by interacting with NIMA and attenuating its mitosis-promoting activity.
*TGFB1*	Transforming growth factor beta-1; Multifunctional protein that controls proliferation, differentiation and other functions in many cell types. It positively and negatively regulates many other growth factors.
*TPX2*	Targeting protein for Xklp2; Spindle assembly factor required for normal assembly of mitotic spindles. It mediates AURKA localization to spindle microtubules. Activates AURKA by promoting its autophosphorylation at ‘Thr-288’ and protects this residue against dephosphorylation.

### Validation of Transcriptomics Analysis

**Figure 5:**
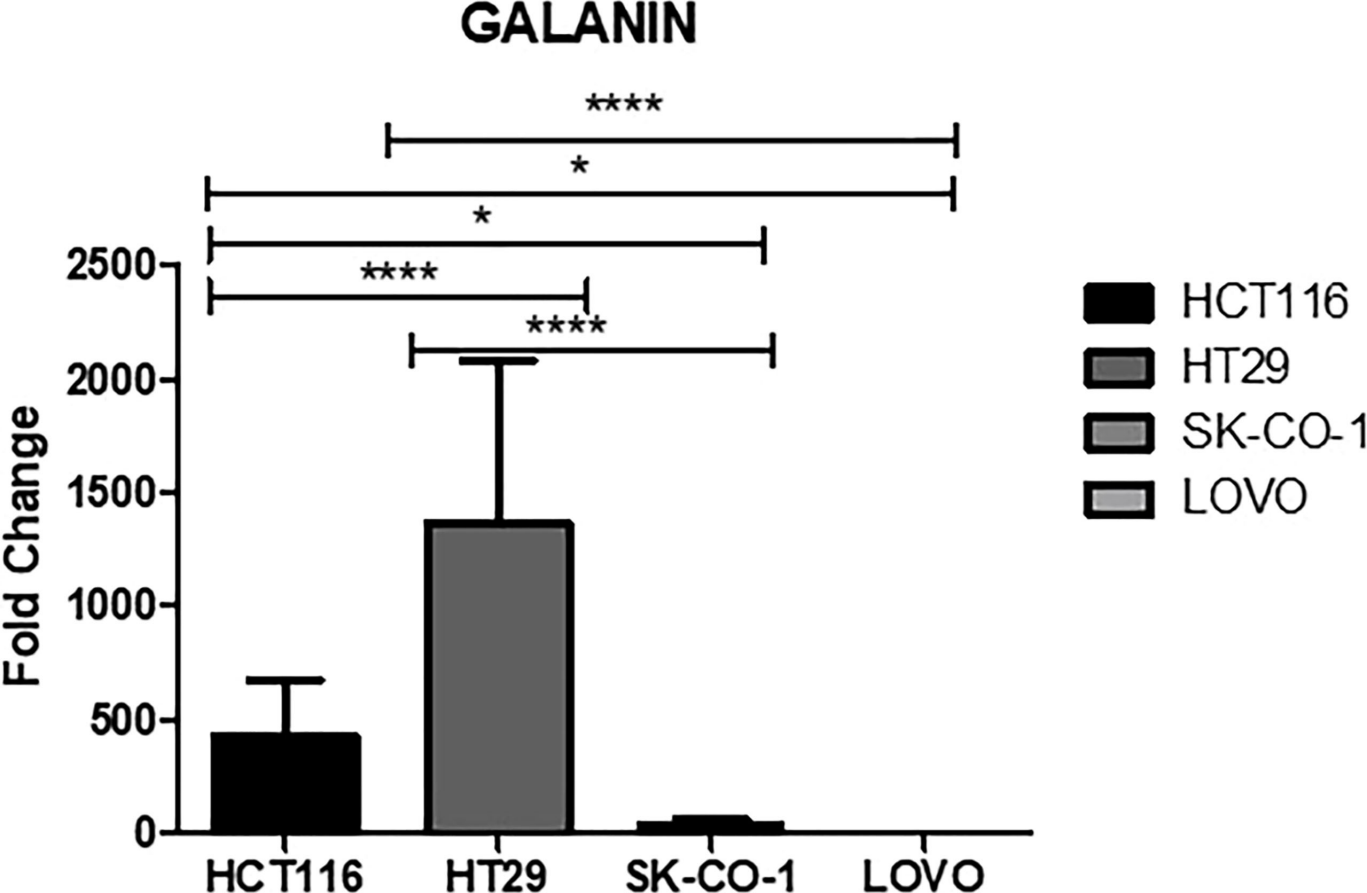
Galanin expression in cell lines. Metastatic cell lines; LoVo and SK-Co-1 showed significantly lower expression of galanin compared to HCT116 and HT29 cell lines. The cells were starved overnight to ensure synchronization. Gene expression in each cell line was normalized to its corresponding non-starved cell line. * denotes p-value <0.05, **** p-value <000.1.

**Figure 6:**
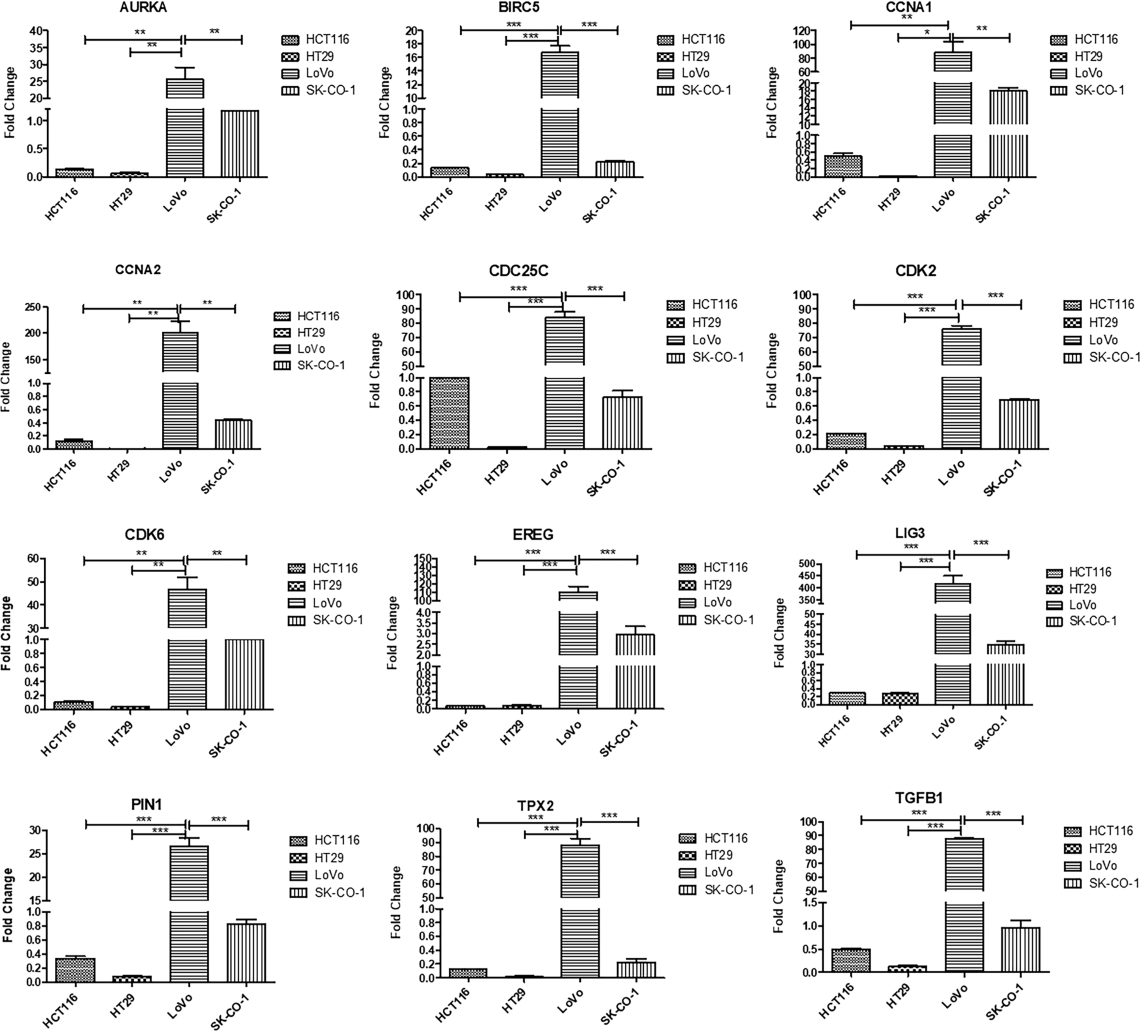
Validation of top upregulated genes in metastatic CRC compared to earlier stages, using Real-time PCR for four cell lines; HCT116, HT29, LoVo and SK-Co-1. The cells were starved overnight to ensure synchronization. Gene expression in each cell line was normalized to its corresponding non-starved cell line. One way ANOVA was used to compare the expression among different cell lines. Metastatic cell lines; LoVo and SK-Co-1 showed significantly higher expression of *AURKA, BIRC5, CCNA1, CCNA2, CDK2, CDK6, EREG, LIG3, PIN1, TGFB1, TPX2*, compared to their expression in HCT116 and HT29. In contrast, CDC25C was also highly expressed in HCT116. * denotes p-value <0.05, ** denotes p-value <0.01, *** denotes p-value <0.001.

Galanin expression was significantly higher in two primary cell lines (HCT116 and HT29) compared to the two metastatic CRC cell lines (LoVo and SK-Co-1), **Figure 5**. We also examined the expressions of the top 12 DEGs shared among different pathways. Results were concordant with the transcriptomics analysis of patients’ samples (**Figure 6**).

### Bioinformatics Analysis

**Figure 7:**
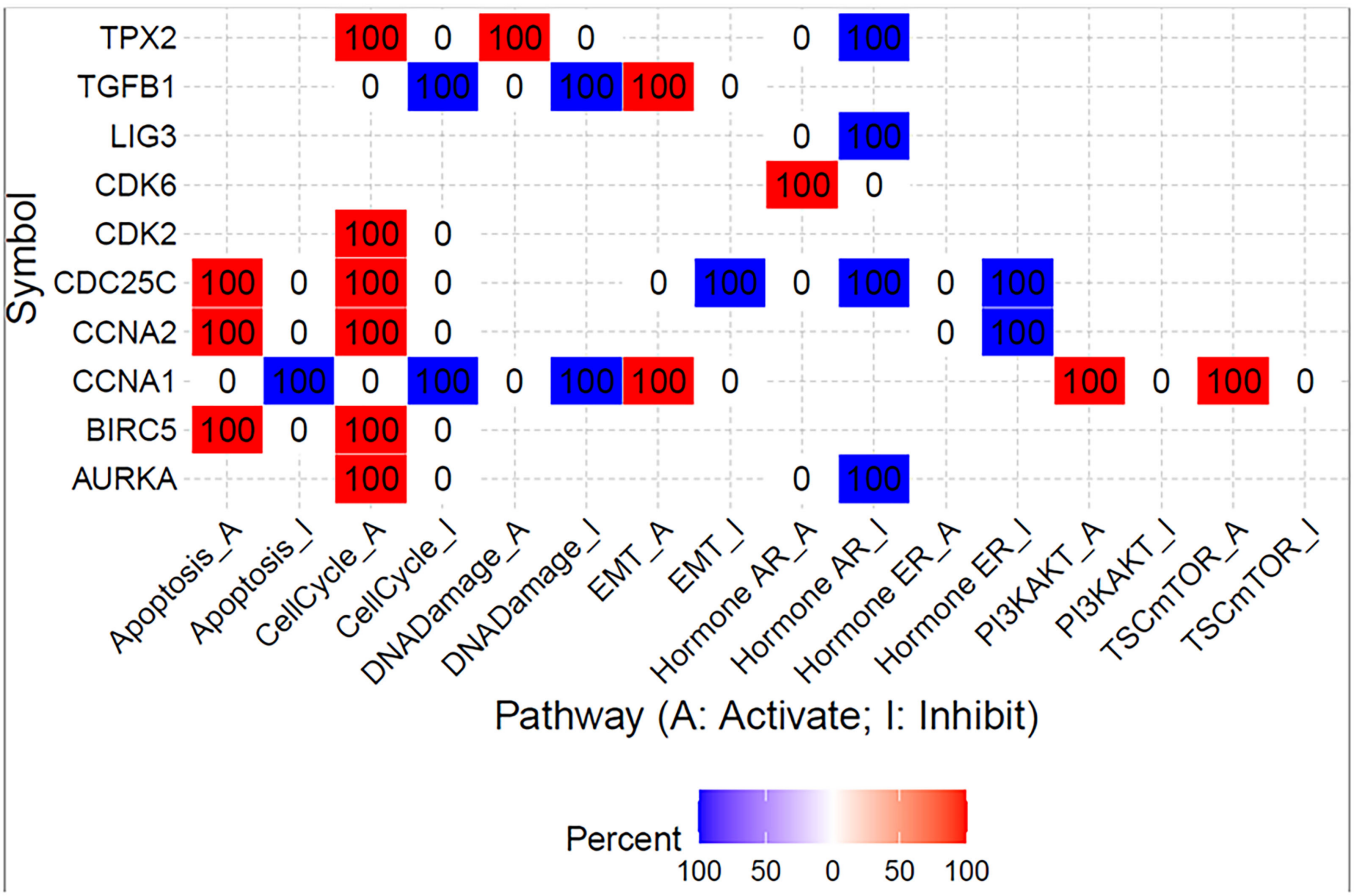
Summary of the percentage of colon adenocarcinoma in which shared gene expression has a potential effect on pathway activity. The analysis was created using Gene Set Cancer Analysis (GSCALite) web tool and TGCA PanCancer COAD dataset.

To further evaluate the effect of galanin expression in CRC, we performed bioinformatics analysis. We assessed the effect of shared genes on the activation or inhibition of cancer-associated pathways using Gene Set Cancer Analysis (GSCALite). **Figure 7** shows the percentage of colon adenocarcinoma cases in which shared gene expression activated (red) or inhibited (blue) specific pathways. Interestingly, there was a consistency in the expression of those genes in all studied CRC transcriptomes. **Supplementary Table S4** enlists the false discovery rate for each shared gene.

**Figure 8:**
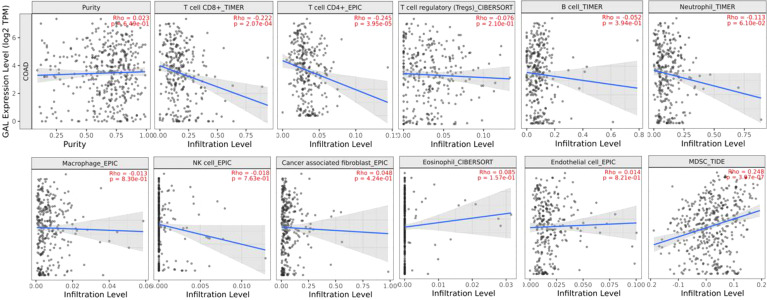
Spearman’s rank correlation coefficient of immune-infiltrating-cell abundance and GAL expression in COAD, using TIMER 2 web tool.

Then, Spearman’s rank correlation coefficient was used to investigate the correlation between galanin expression and the immune-infiltrating-cell abundance in CRC using the TIMER 2 web tool. There was a negative correlation between the galanin expression and T cell CD8+, CD4+, neutrophil and natural killer (NK) cell numbers in the COAD tumor microenvironment (TME). However, there was a positive correlation between galanin expression and myeloid-derived suppressing cells (MDSC), denoting its association with a “non-inflamed” TME (cold TME), **Figure 8**.

Interestingly, galanin expression is significantly lower in CRC vs normal colonic mucosa (**Supplementary Figure S9**). The highest expression of galanin was found in the age group of 21-40 years compared to other age groups (**Supplementary Figure S10**).

## Discussion

The current study highlights the significance of galanin downregulation in CRC progression. In our study, galanin showed significantly lower expression in patients’ samples of stage IV compared to earlier stages, evident by immunohistochemical staining. Although the galanin intensity is generally weakly correlated with TNM staging, galanin downregulation was associated with differential expression of key genes and driver pathways related to CRC progression, as revealed by our transcriptomics analysis. This was validated in the more controlled cell line experiments that showed a significant increase in the expression of galanin in the primary cell lines compared to the metastatic ones. In addition, the cell line experiments validated the results of differential expression of the transcriptomics analysis.

There is a complex interaction between the tumor and its microenvironment, leading eventually to further cancer cells’ proliferation, invasion, and inhibition of apoptosis. Interestingly, it was reported that the number of galanin-containing neurons within myenteric plexuses located in the vicinity of the infiltrating cancer was higher in comparison to distal marginal regions (25). As a result of the invasion, nerve fibres of the enteric nervous system are destroyed, with subsequent atrophy of the myenteric plexus and the submucosal plexus. In the current study, in stage II CRC, galanin expression was at the highest in cancer cells, consistent with the previous findings of Nagayoshi et al. (12). With further progression, galanin expression declined, reaching its minimum in stage IV, where most neuronal plexuses are expected to be completely atrophied. However, this is controversial since galanin was reported in a previous study as a neuroprotective peptide that may inhibit the extrinsic pathway of apoptosis and, subsequently, promote cancer cell survival (26).

In the current study, transcriptomics analysis of representative samples of early-stage (TNM stage I) and late stages (stages II and III) identified five key pathways as drivers for tumor progression from early to late stages. Differentially upregulated pathways included cell cycle, cell division, autophagy, transcriptional regulation of *TP53* and immune system process. We identified twelve shared genes among different upregulated pathways and validated the results using Real-time PCR on cell lines.

Late stages of CRC in our study showed upregulation of the cell cycle and cell division pathways. One of the key characteristics of cancer is the uncontrolled proliferation due to derangement of cell cycle control through major checkpoints at G1–S transition and at G2–M transition (22, 23). Furthermore, autophagy plays an essential role in the progression of CRC under the increased mutational burden or through oncogenes or tumor suppressor genes. However, it is still doubtful if autophagy is an anti- or pro- cancerous (27). It was observed that expression of LC3B and SQSTM1 was associated with CRC’s poor prognosis (28). In another study, although down-regulation of autophagy-related gene-5 (*ATG5*) was detected, its expression was associated with lymphovascular invasion (16). It was found that CRC with high microsatellite instability harbored at least one mutation in either *ATG12, ATG 9B, ATG 5, or ATG 2B* (29). In a study conducted by Lévy et al., depletion of ATG 7 in intestinal cells of mouse models was found to hinder cancerous growth (30). A gene signature related to autophagy was previously identified to group colon cancer patients into high or low risk in order to predict survival (31). Noteworthy, *TP53* mutations occur very frequently in CRC and are known to drive the progression from adenoma to adenocarcinoma (32). The TP53 can harness autophagy by degrading autophagy protein LC3 in CRC cell lines. It was also found that loss of TP53 promoted the accumulation of LC3 initiating apoptosis (33).

The immune system process pathway is one of the differentially upregulated ones in late CRC samples in our study. Immune cells play a pivotal role in shaping the TME and determining the tumor progression (34), modulating inflammation (35), and metastasis (36). Thus, the TME, with its immune-cancer interaction, has a significant impact on the diagnosis and treatment of different malignancies, including CRC (37). The cellular, genetic, and molecular characteristics of the TME may affect the response to immune checkpoint inhibitors (38). In addition, chemotherapeutic agents used to treat CRC may also alter the expression of key molecules in the TME, e.g., capecitabine was recently reported to suppress the expression of Cytotoxic T lymphocyte antigen-4 (CTLA-4) in CRC, with potential enhancement of immunotherapy (39).

To further explore the relationship between galanin expression and the different immune cells in the TME, we conducted bioinformatics analysis. The analysis revealed a negative correlation of galanin expression with the number of several tumor-infiltrating immune cells, including CD8+ and CD4+ T-cells, neutrophils, and natural killer cells in the TME. In contrast, the galanin expression was positively correlated with the number of MDSC. Such findings denote the association of galanin expression with “cold” or “less inflamed tumors” with a poorer prognosis. They also represent a significant challenge of immunotherapy in different types of cancer, as there is a lack of adequate adaptive immune response (40).

Our results showed downregulation in the late stages of CRC of pathways involved in neuroactive ligand-receptor interaction compared to early stages. A neuroactive ligand-receptor interaction pathway was previously reported among the ones for which miRNAs were enriched in a study comparing responders and non-responders in terms of sensitivity to preoperative chemoradiotherapy for rectal cancer (41). Similarly, the neuroactive ligand-receptor interaction pathway was significantly different between colon tumor tissues and the adjacent noncancerous tissues using microarray data (42, 43).

We also examined the potential role of the top shared genes among the upregulated pathways, namely, *AURKA, BIRC5, CCNA1, CCNA2, CDC25C, CDK2, CDK6, EREG, LIG3, PIN1, TGFB1*, and *TPX2.* Collectively, the upregulation of those genes activates the cell cycle, EMT, PI3K/AKT and mTOR pathways.

Our bioinformatics analysis revealed some interesting information about galanin; galanin expression is significantly lower in CRC vs normal colonic mucosa (consistent with our results). The highest expression occurs in CRC patients in the age group of 21-40 years. Though sporadic CRC is usually detected after the sixth decade of life, there has been an increase in CRC incidence in patients under the age of 40. Different molecular characteristics and a low suspicion of CRC in symptomatic young persons may underlie this incidence (44). Galanin expression may be linked to the early stage at which the disease is diagnosed at this young age group.

The current study has a few limitations, including the small patient cohort, with under-represented stages I and IV. In addition, we limited our transcriptomics analysis to the non-metastatic CRC patients’ samples, considering the complexity and diversification of the biological programs underlying the metastatic CRC. Our current study mandates further biological validation to evaluate the different pathways detected by the transcriptomics analysis in association with low galanin expression. However, the current study is the first to show the role of galanin in CRC progression at the molecular level in one of the Northern African populations. The transcriptomics analysis links galanin downregulation with key genes involved in key cancer-associated pathways.

## Conclusions

There is a negative correlation of galanin intensity with CRC progression, being expressed most intensely in stage II and least in stage IV. Key driver pathways of tumor progression were revealed *via* transcriptomics analysis of late versus early-stage CRC tissue samples. The pathways included cell cycle and division, autophagy, transcriptional regulation of TP53 and immune system process.

The top shared genes among the upregulated pathways are *AURKA, BIRC5, CCNA1, CCNA2, CDC25C, CDK2, CDK6, EREG, LIG3, PIN1, TGFB1*, and *TPX2.* Interestingly, those genes are members of the cell cycle and cell division pathways. Moreover, BRIC5 is a member of the inhibitor of the apoptosis gene family. Galanin could represent a negative biomarker of CRC progression. Shared genes and pathways in late versus early stages of CRC provide insight into the progression of the disease and may serve as early biomarkers of metastatic disease.

## Data Availability Statement

The datasets presented in this study can be found in online repositories. The names of the repository/repositories and accession number can be found at: https://doi.org/10.6084/m9.figshare.19895779.

## Ethics Statement

The studies involving human participants were reviewed and approved by Research Ethics Committee, University of Sharjah. Written informed consent for participation was not required for this study in accordance with the national legislation and the institutional requirements.

## Author Contributions

IT: Conception, interpretation of data, preparation of the manuscript, supervision, MS-A: Conception, data analysis, preparation of the manuscript, supervision, NMY: data analysis, AS: data analysis, interpretation of data, TV: data analysis, Transcriptomics analysis and qPCR. AV: data acquisition, sample processing and preparation, AH: data acquisition, AK: clinical data acquisition, WA-R: interpretation of data, revising the manuscript, RH: conception, supervision, revising the manuscript. All manuscript authors agreed on the aforementioned contribution and have revised and approved the submitted version.

## Funding

This research was funded by Al-Jalila Foundation Seed Grant (#AJF2017-68), MBRU-AlMahmeed Research Award, 2019 (ALM#1914).

## Conflict of Interest

The authors declare that the research was conducted in the absence of any commercial or financial relationships that could be construed as a potential conflict of interest.

## Publisher’s Note

All claims expressed in this article are solely those of the authors and do not necessarily represent those of their affiliated organizations, or those of the publisher, the editors and the reviewers. Any product that may be evaluated in this article, or claim that may be made by its manufacturer, is not guaranteed or endorsed by the publisher.
