# Supplementary material for: Evaluation of Galanin Expression in Colorectal Cancer: An Immunohistochemical and Transcriptomic Study
Source: Front Oncol. 2022 May 30;12:877147. doi: 10.3389/fonc.2022.877147 (PMC9190230; doi:10.3389/fonc.2022.877147)
Supplement: Supplementary file 1 [file DataSheet_1.docx]

Supplementary Material:

Appendix A: Supplementary Material

Table S1: The clinicopathological parameters in our patient cohort (81 CRC patients)

Table S2: Age correlation with different clinicopathological parameters

Table S3: Comparison of galanin intensity scores in CRC of different TNM stages

Table S4: Differences in pathway activity between high and low mRNA expression of each of the top 12 DEG.

Figure S1: Figure S1. Representative images of immunohistochemical staining of Galanin Receptor 1 (GALR1) in patients’ samples.

Figure S2: Enrichment score plot for R-HAS-1640170: Cell cycle

Figure S3: Enrichment score plot for GO:00066914: Autophagy

Figure S4: Enrichment score plot for GO:0051301: Cell Division

Figure S5: Enrichment score plot for GO: 0002376: Immune system process

Figure S6: Enrichment score plot for R-HAS-3700989: Transcriptional Regulation by TP53

Figure S7: Leading subsets assignment

Figure S8: Leading subsets overlap

Figure S9: Galanin expression in primary colon cancer versus normal

colonic tissue

Figure S10. Expression of galanin in normal and colon adenocarcinoma

(COAD) based on different age groups.

Supplementary Tables

**Supplementary Table S1**. The clinicopathological parameters in our patient cohort (81 CRC patients)

| **Clinicopathological Parameters** | **Number** | **%** |
| --- | --- | --- |
| **Site** |  | |
| - Right | 35 | 43.2% |
| - Left | 46 | 56.8% |
| **Grade*** |  | |
| - Grade I | 5 | 6.25% |
| - Grade II | 66 | 81.4% |
| - Grade III | 9 | 11.1% |
| **Stage** |  | |
| - Early (TNM stage I) | 14 | 17.3% |
| - Late (TNM stage II and III) | 61 | 75.3% |
| - Advanced (stage IV) | 6 | 7.4% |

*One case diagnosed as mucinous CRC, not graded based on the degree of glandular differentiation.

Supplementary Table S2. Age correlation with different clinicopathological parameters

| **Parameter** | **Correlation with Age** | |
| --- | --- | --- |
|  | **Sig. (2-tailed)** | **Pearson Correlation** |
| Max Diameter | 0.365 | 0.102 |
| TNM staging  (I- IV) | 0.063 | -0.207 |
| Stage  (Early versus late) | 0.006* | -.305** |
| Galanin intensity | 0.196 | 0.145 |

Age showed a significantly negative correlation with the disease stage (significance appeared in classifying the patients into two groups: early and late, p-value of 0.006). Early-stage (TNM stage I) and late-stage (TNM stages II and III)

**Supplementary Table S3.** Comparison of galanin intensity scores in CRC of different TNM stages

| **Galanin Intensity** |  | **0** | **1** | **2** | **3** | **Total** |
| --- | --- | --- | --- | --- | --- | --- |
| TNM Staging | 1 | 2 | 7 | 4 | 1 | 14 |
|  | 2 | 7 | 12 | 5 | 9 | 32 |
|  | 3 | 9 | 6 | 13 | 0 | 28 |
|  | 4 | 3 | 3 | 0 | 0 | 6 |
| Total | | 21 | 28 | 22 | 10 | 81 |

There is a significant difference in galanin intensity according to the TNM staging (p-value = 0.005 using Pearson Chi-Square).

**Supplementary** **Table S4.** Differences in pathway activity between high and low mRNA expression of each of the top 12 DEG.

| **Gene Symbol** | **Pathway** | **Class** | **FDR** |
| --- | --- | --- | --- |
| AURKA | CellCycle | Activation | 0.000881 |
| BIRC5 | Apoptosis | Activation | 0.000133 |
| BIRC5 | CellCycle | Activation | 2.09E-07 |
| CCNA1 | EMT | Activation | 2.51E-06 |
| CCNA1 | PI3KAKT | Activation | 0.000784 |
| CCNA1 | TSCmTOR | Activation | 0.02917 |
| CCNA2 | Apoptosis | Activation | 0.000743 |
| CCNA2 | CellCycle | Activation | 5.82E-07 |
| CDC25C | Apoptosis | Activation | 0.018964 |
| CDC25C | CellCycle | Activation | 4.77E-08 |
| CDK2 | CellCycle | Activation | 2.85E-06 |
| CDK6 | Hormone AR | Activation | 0.001234 |
| TGFB1 | EMT | Activation | 1.67E-06 |
| TPX2 | CellCycle | Activation | 7.47E-05 |
| TPX2 | DNADamage | Activation | 0.027974 |
| AURKA | Hormone AR | Inhibition | 0.002095 |
| CCNA1 | Apoptosis | Inhibition | 0.000761 |
| CCNA1 | CellCycle | Inhibition | 6.12E-05 |
| CCNA1 | DNADamage | Inhibition | 0.042051 |
| CCNA2 | Hormone ER | Inhibition | 0.019323 |
| CDC25C | EMT | Inhibition | 0.001581 |
| CDC25C | Hormone AR | Inhibition | 0.035577 |
| CDC25C | Hormone ER | Inhibition | 0.025274 |
| LIG3 | Hormone AR | Inhibition | 0.040392 |
| TGFB1 | CellCycle | Inhibition | 0.014352 |
| TGFB1 | DNADamage | Inhibition | 0.000577 |
| TPX2 | Hormone AR | Inhibition | 0.027974 |

FDR is “False discovery rate”, it can be considered as an adjusted p-value to trim false-positive results.

**Supplementary Figures**

**
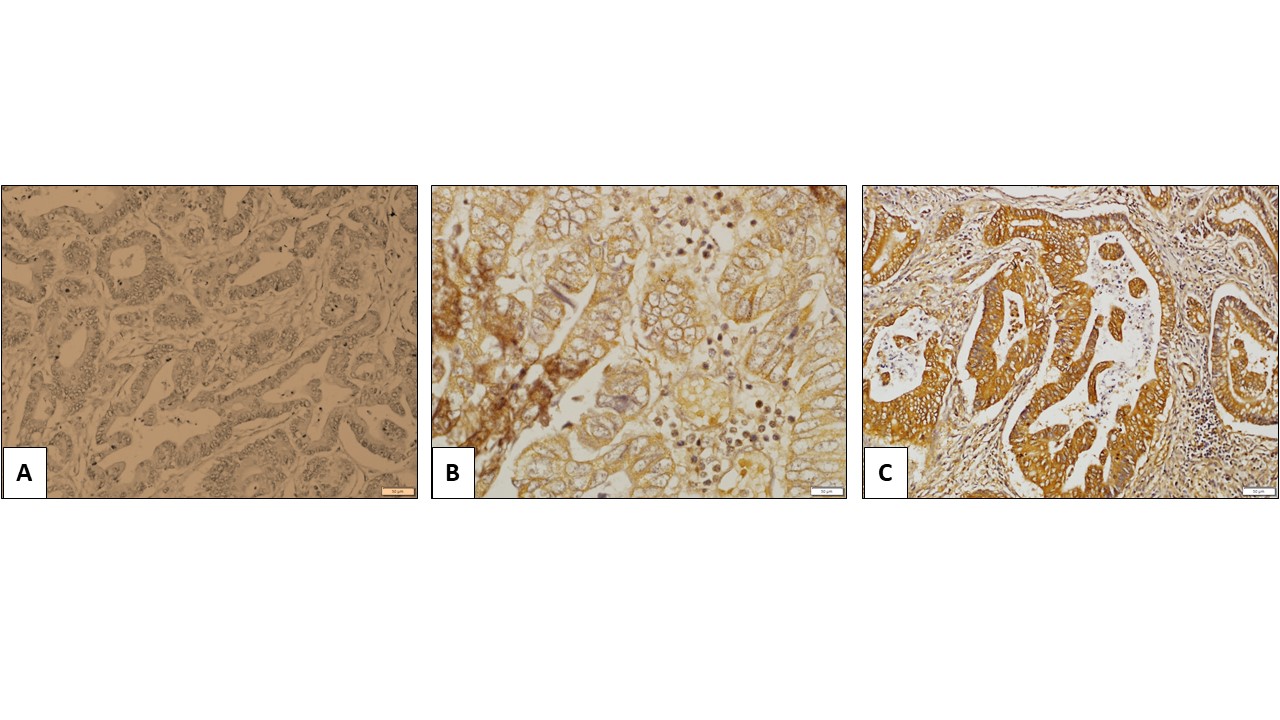
**

**Figure S1A.** Images of immunohistochemical staining of Galanin Receptor 1 (GALR1) in patients’ samples. Staining by immunoperoxidase, scale bar: 50μm. A. Mild expression B. Moderate expression C. High expression.


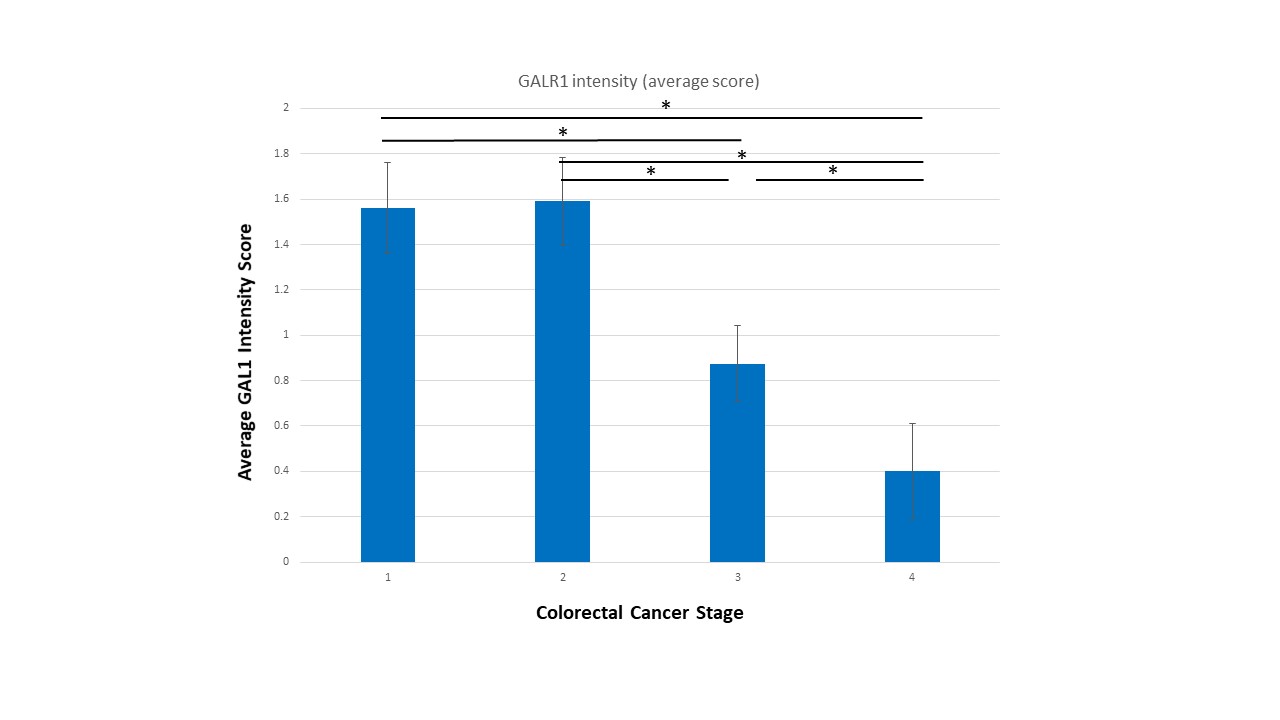


**Figure S1B.** GALR1 intensity according to TNM staging. GALR1 intensity is significantly lower in stage IV CRC compared to stage I-III, * p-value <0.05 (using the non-parametric Mann-Whitney U test).


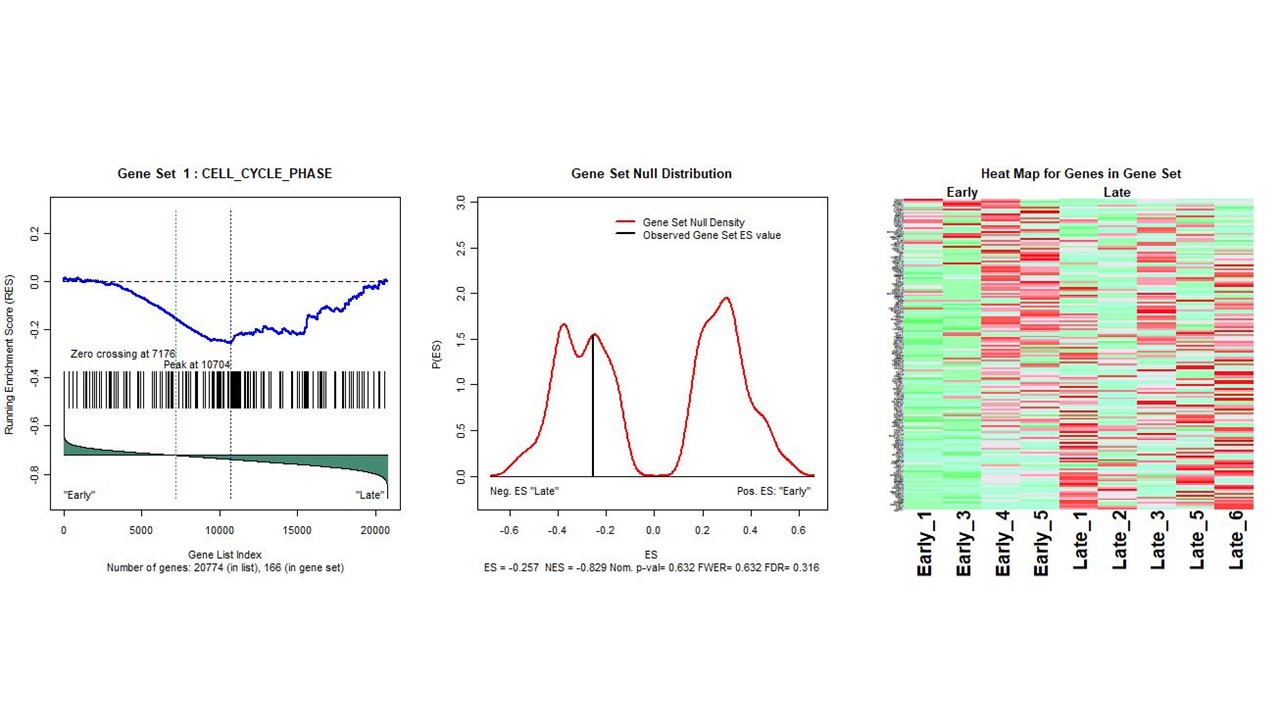


Figure S2: Enrichment score plot for R-HAS-1640170: Cell cycle.


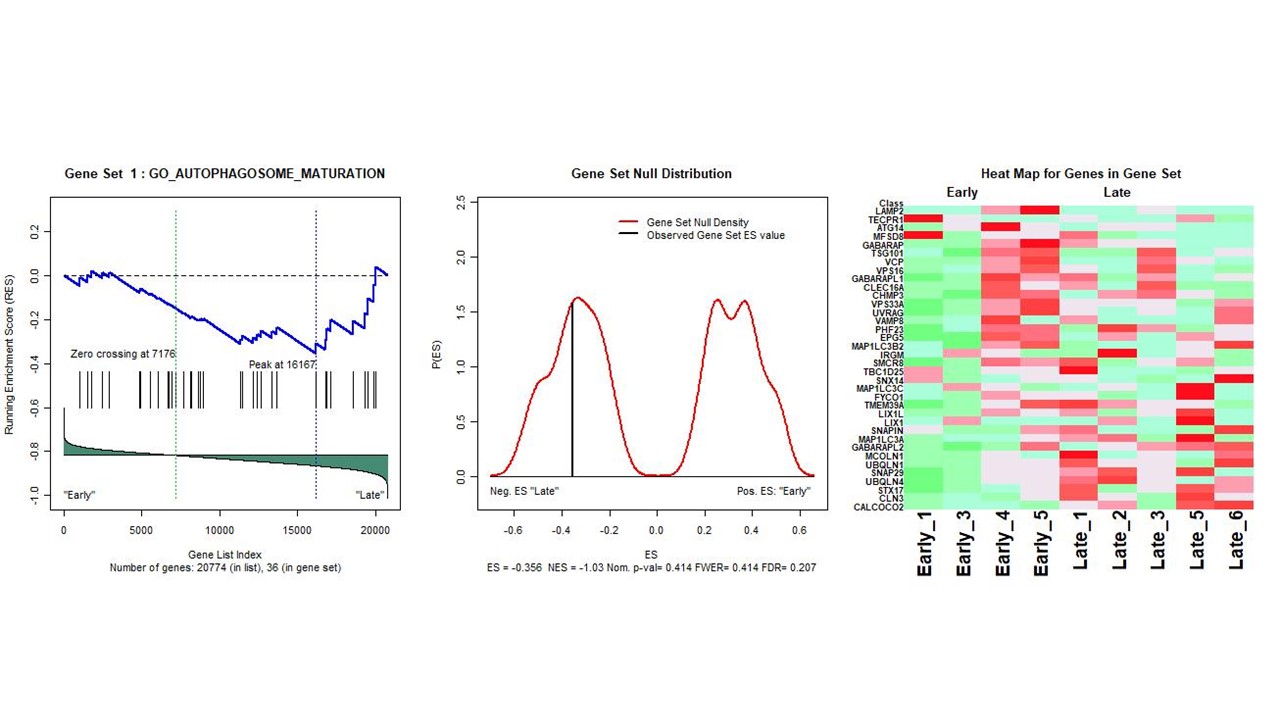


Figure S3: Enrichment score plot for GO:00066914: Autophagy.


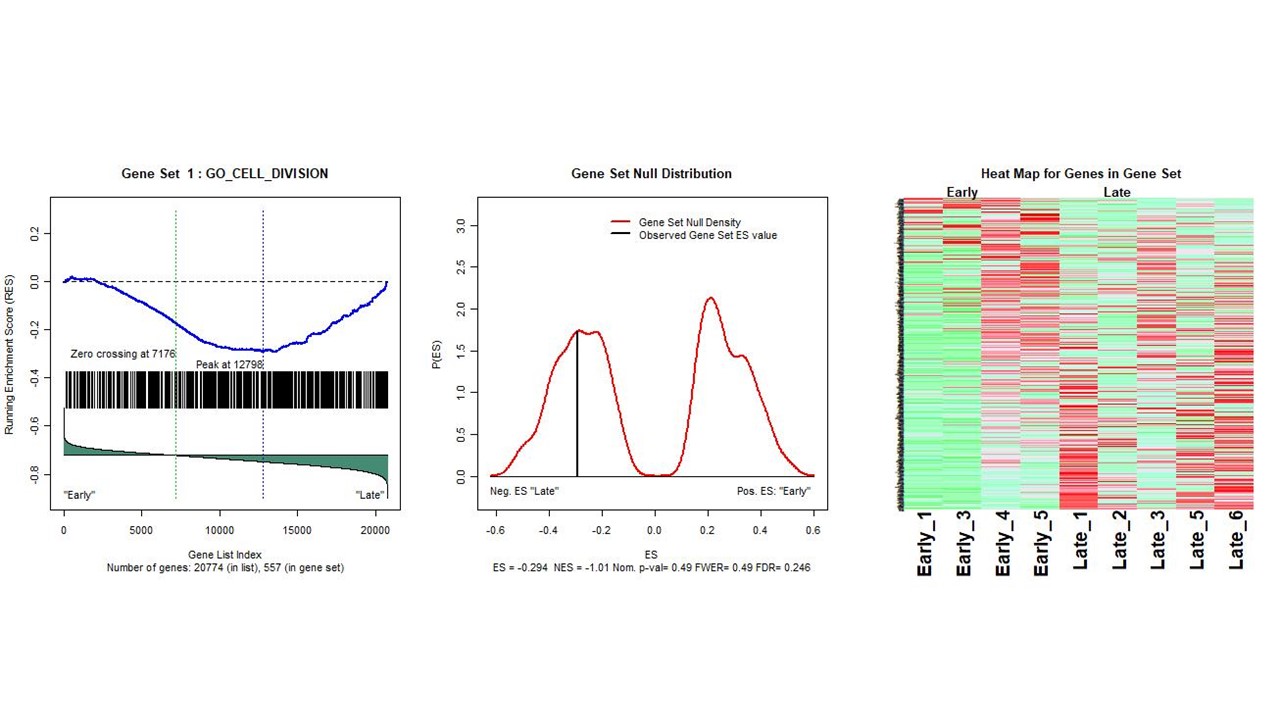


Figure S4: Enrichment score plot for GO:0051301: Cell Division.


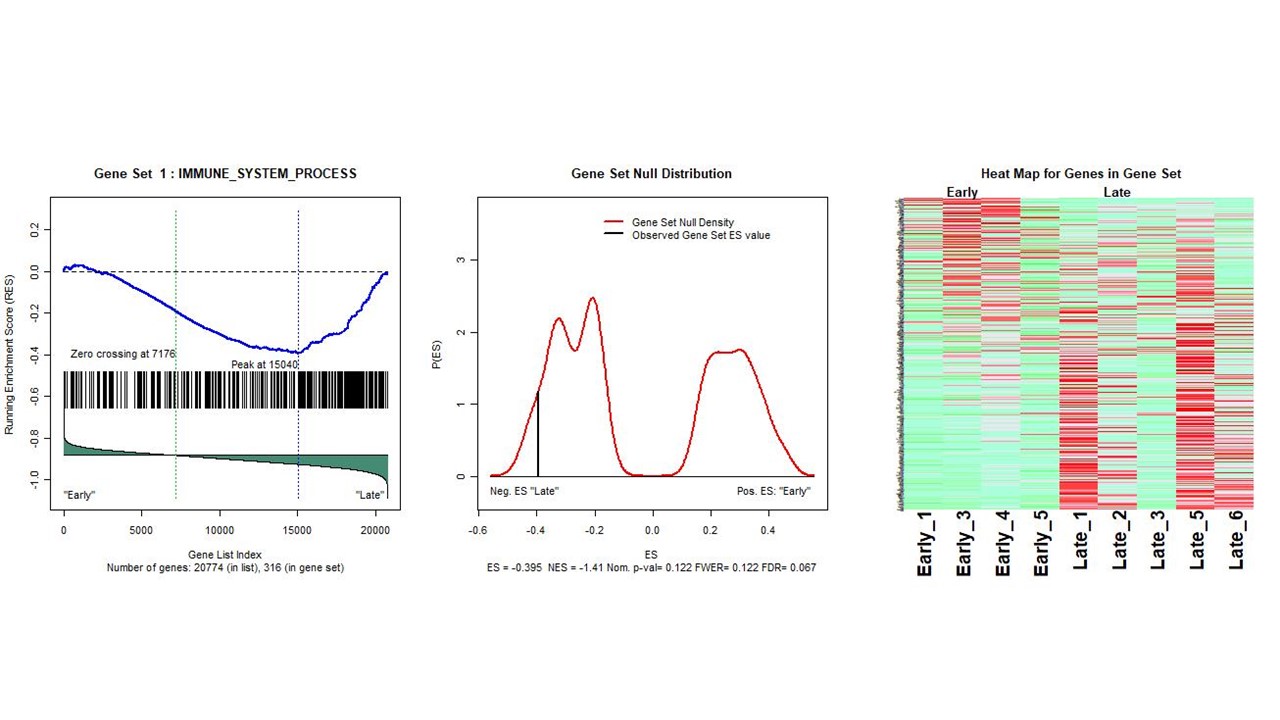


Figure S5: Enrichment score plot for GO: 0002376: Immune system process.


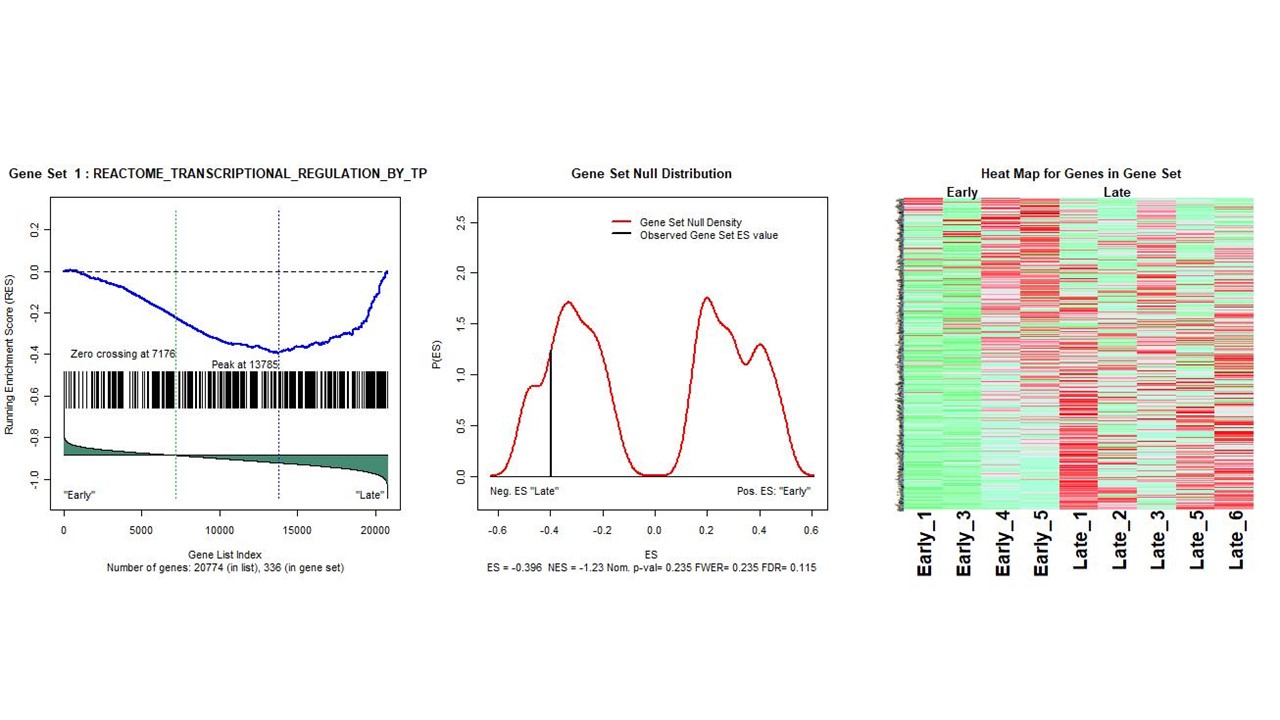


Figure S6: Enrichment score plot for R-HAS-3700989: Transcriptional Regulation by TP53.


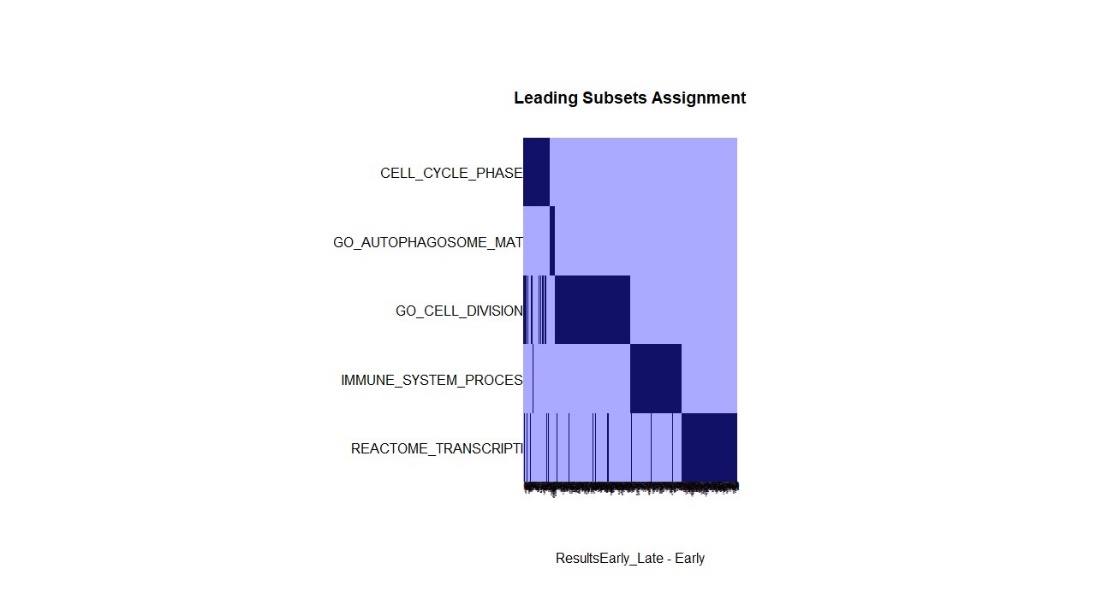


Figure S7: Leading subsets assignment.


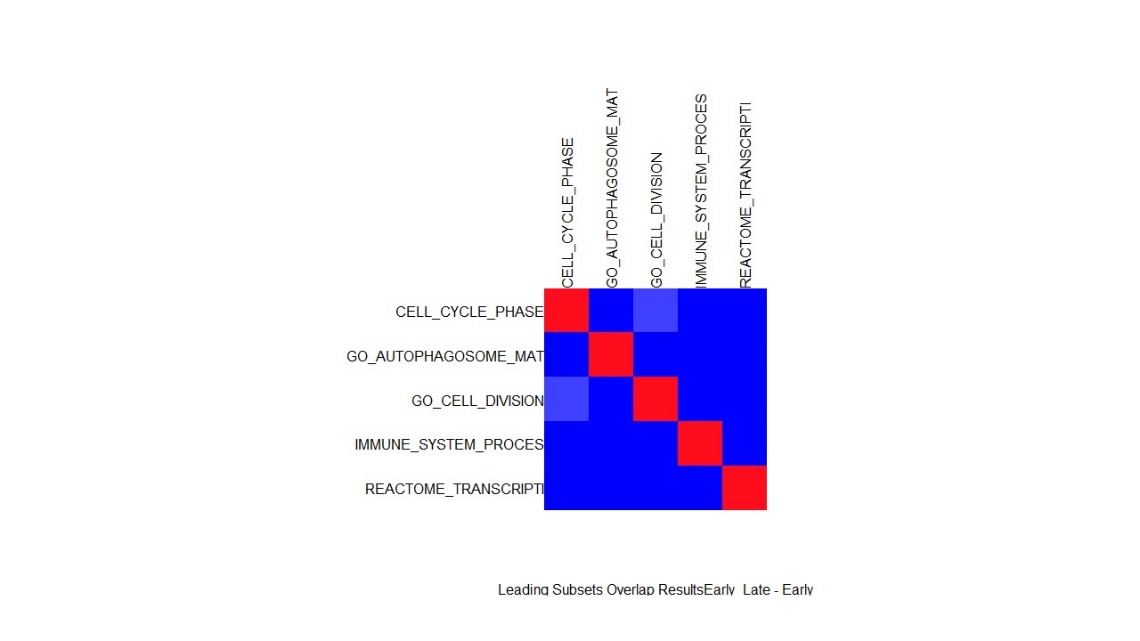


Figure S8: Leading subsets overlap.


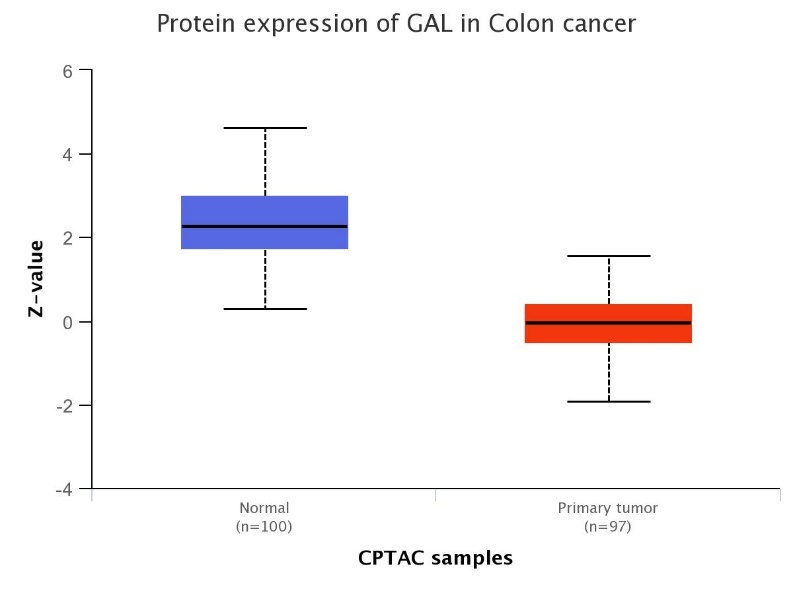


**Figure S9:** Galanin expression in primary colon cancer versus normal colonic tissue, using the CPTAC dataset and UALCAN web tool. Galanin expression is significantly lower in colon cancer (n=97) vs normal colonic mucosa (n=100).


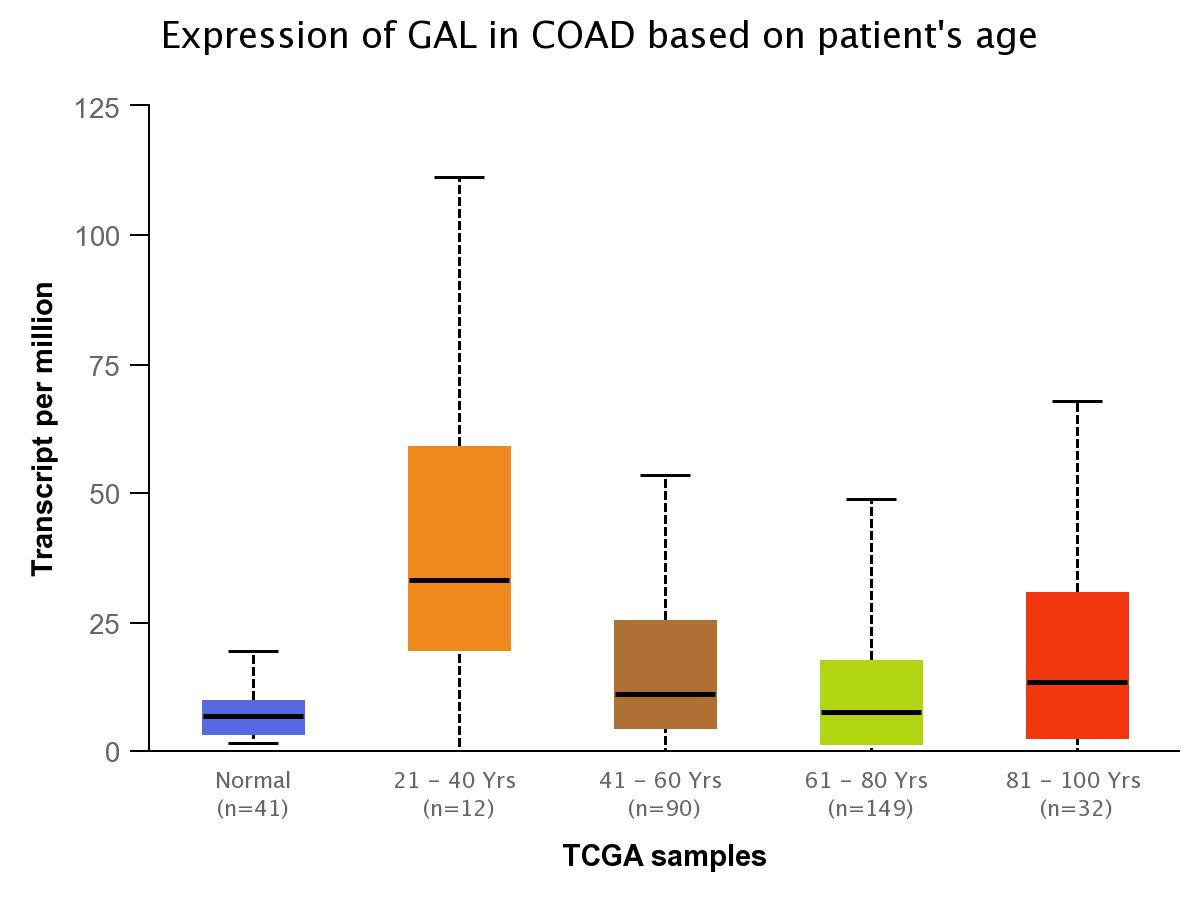


**Figure S10.** Expression of galanin in normal and colon adenocarcinoma (COAD) based on different age groups. The highest expression of galanin was found in the age group of 21-40 years (n=12).
